# The Reliability of Histamine Pharmacodynamic Response Phenotype Classification in Children With Allergic Disease

**DOI:** 10.3389/fphar.2020.00227

**Published:** 2020-03-11

**Authors:** Shaun S. Kumar, Xiaoxi Liu, Catherine M. Sherwin, Bridgette L. Jones

**Affiliations:** ^1^Division of Clinical Pharmacology, Department of Pediatrics, University of Utah School of Medicine, Salt Lake City, UT, United States; ^2^Department of Pharmacotherapy, College of Pharmacy, University of Utah, Salt Lake City, UT, United States; ^3^Department of Pediatrics, Dayton Children’s Hospital, Wright State University Boonshoft School of Medicine, Dayton, OH, United States; ^4^Division of Pediatric Pharmacology, Therapeutic Innovation, Children’s Mercy Hospital, Kansas City, MO, United States; ^5^Division of Allergy, Asthma, Clinical Immunology, Children’s Mercy Hospital, Kansas City, MO, United States; ^6^Department of Pediatrics, University of Missouri, Kansas City, MO, United States

**Keywords:** histamine, children, pharmacodynamics, allergy, phenotype

## Abstract

We have identified distinct histamine pharmacodynamic response phenotypes in children with allergic disease utilizing histamine iontophoresis with laser Doppler (HILD). These response phenotypes may be relevant in guiding therapeutic decision making for agents targeting the allergic response pathways. However, the reliability of these response phenotypes has not been assessed. Therefore, we performed HILD in children with allergic rhinitis and/or asthma on two to three separate occasions. HILD response-time data were analyzed in NONMEM using a linked effect PKPD model. Examination of observed vs. classified response phenotypes predicted response plots and the sum of residuals. The intraclass correlation coefficient (ICC) was used to determine the reliability of phenotype classification. Eighty-two percent of children exhibited a reliable histamine response phenotype [intraclass correlation coefficient 0.77 (95% CI 0.44–0.93]. These preliminary results suggest moderate reliability of HILD response phenotype in children. Further exploration is needed to determine contributions to phenotype variability.

## Introduction

Histamine is a small molecule that is released by various cells throughout the body. The amine is most commonly associated with the allergic response whereby when binding to the Histamine-1 receptor (H1R) it sets off a cascade of responses that lead to itching, sneezing, mucous production, and bronchoconstriction. However, recently histamine has been discovered to have pervasive effects throughout the body which include the respiratory system, gastrointestinal tract, central nervous system, bone marrow, cardiovascular system, genitourinary system, as well as immunoregulatory effects (Huang and Thurmond, 2008; Thurmond et al., 2008). Due to the broadened understanding of histamine, it has been suggested that the amine plays a more significant role in the pathogenesis of diseases such as asthma and atopic dermatitis than previously believed.

Asthma is a heterogeneous disease of various phenotypes (e.g., allergic asthma vs. non-allergic asthma) in addition to variability in pathophysiology among more defined disease phenotypes (e.g., multi-allergen sensitivity vs. limited allergen sensitivity). Evidence suggests that some patients with specific asthma phenotypes may benefit from anti-allergic treatment in the management of the disease more than others (Warner and ETAC Study Group, 2001). However, biomarkers are needed to determine which patients may benefit most from the addition of anti-allergic/antihistamine treatment. The epicutaneous histamine “skin prick” test is the “gold standard” method for the clinical evaluation of allergic conditions and is utilized in clinical trials to assess antihistamine pharmacodynamic response (Deschamps et al., 2000; Simons and Simons, 2005). The epicutaneous histamine “skin prick” test involves manually delivering histamine to the epicutaneous surface of the skin via “prick” device and the response to histamine is assessed by utilizing a visual measuring device (e.g., caliper device) to determine the size of the wheal and flare response after a given period of time. This method is limited in providing an objective assessment of histamine pharmacodynamic response. These limitations include (1) intra-operator and device variability in histamine delivery, (2) inability to administer a fixed dose of histamine, (3) operator subjectivity in assessing the wheal and flare response, (4) inability to continuously assess response, and (5) somewhat invasive/irritating nature of the procedure which limits its use in very young children.

Histamine iontophoresis with laser Doppler (HILD) is an alternative method to assess histamine pharmacodynamic response in children and adults that overcomes the stated limitations of the epicutaneous “skin prick” test. HILD allows a fixed dose of histamine to be delivered into the skin non-invasively, and blood flow response is measured via a laser Doppler flowmetry device in an objective, continuous, and dynamic manner. Therefore, this method may be more suitable for characterizing histamine pharmacodynamics response that the epicutaneous skin test procedure.

Previously, we have shown that HILD provides an objective, continuous, and dynamic measurement of histamine response in both adults and children (Jones et al., 2009, 2013). Furthermore, we have demonstrated that there are distinct histamine response phenotypes: Hypo-responsive, Normo-responsive, and Hyper-responsive (Jones et al., 2013). We also observed that histamine pharmacodynamic response is associated with genetic polymorphisms that may augment response at the histamine receptor sites or alter enzymatic degradation of histamine (Jones et al., 2016). Therefore, HILD may be useful in determining differences in the biological response to histamine that may be driven by genetic makeup.

We believe that observed histamine pharmacodynamics response phenotypes may be useful in predicting which children may have a more exaggerated response to histamine and therefore may benefit most from treatment with anti-allergic therapies. However, further validation of this tool is necessary. Therefore, we aimed to determine the intra-individual reliability of observed histamine response phenotypes in children as the next step in validating HILD as a suitable biomarker of histamine pharmacodynamics response.

## Materials and Methods

### Participants

The protocol for this study was approved by the Children’s Mercy Institutional Review Board (IRB # 11120477). Children with allergic rhinitis aged 7–19 years of age were recruited via convenience sampling from the Children’s Mercy Allergy/Asthma/Immunology clinics after obtaining parental permission and where appropriate (i.e., age ≥7 years), child assent or consent.

### HILD

**FIGURE 1:**
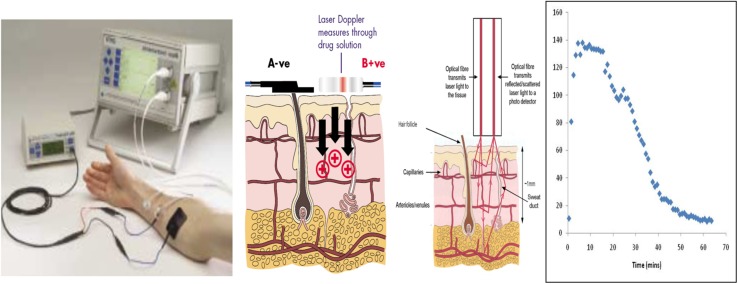
Illustration of the HILD set up. Left most panel – shows the instrumentation set up. Middle left panel – shows the delivery of the drug through the positive terminal. Middle right panel – shows the transmission of the laser into the tissue and the return of the scattered light which is detected by the photo detector. Right panel – shows an example histamine (flux) profile over time. Images in the first two panels provided with permission and courtesy of Moor Instruments.

Histamine iontophoresis with laser Doppler was performed in nineteen participants in identical fashion on 2–3 separate occasions. HILD was conducted following a wash-out period from the use of agents that alter histamine response (e.g., antihistamines – 10 days, systemic steroids – 30 days, and tricyclic antidepressants – 30 days). For iontophoresis and Doppler monitoring, a solid-state, single-frequency laser probe was inserted into the center of an iontophoresis chamber attached to a laser Doppler blood flow monitor (DRT4, Moor Instruments Ltd., Wilmington, DE, United States) and placed on the volar surface of the forearm. A second laser Doppler control probe placed at a distance of at least 1 cm from the iontophoresis site. 200 μL of histamine dihydrochloride solution (1%; Sigma Chemical Ltd., Dorset, United Kingdom), dissolved in a 2% methylcellulose gel (Sigma-Aldrich Chemical Co., St. Louis, MI, United States), was placed in the reservoir of the iontophoresis chamber. For iontophoresis, constant anodal current (50 μA) was applied and values for small vessel blood flow at each of the probe sites were calculated by accompanying software (Moor Instruments Ltd., Devon, United Kingdom) and were expressed in perfusion units (flux, photocurrent produced by the scattering of light by moving red blood cells). Baseline blood flow was assessed for 2 min before the iontophoresis procedure. Blood flow was monitored continuously until measurements returned to baseline or for a maximum of 2 h. A more detailed description of the HILD test has been previously published (Jones et al., 2009, 2013). An example of the HILD setup is provided in Figure 1.

### Data Handling

A total of 26 HILD tests were available to assess reliability. Each response-time profile was examined individually. Of the 19 participants, 11 participants had evaluable data for more than one HILD test. Four participants had usable profiles for three tests, and the remaining patients had usable profiles for two tests. Flux measurements were sampled at 50 Hz for up to 2 h in each participant who produced a maximum of 3600 data points per profile. To allow for easier handling for pharmacometric software, the data were paired down as previously described in detail (Liu et al., 2016). Briefly, the data were divided evenly into 200-time segments. The response data were then averaged within each segment, and the median time of each segment was used. Finally, 10–15 data points per profile were selected that provided a realistic representation of the response-time profile. These reduced data were then used for pharmacometric analysis.

### Model Development

The response-time data were analyzed using NONMEM v7.3 (ICON Development Solutions, Ellicott City, MD, United States). Pearl-speaks-NONMEM (PsN) 4.4.8 and Pirana 2.9.6 were used to facilitate model development. A previously published direct linked PK/PD model based on 156 children (single tests) (Liu et al., 2016) was used initially to guide model development. The model was a one-compartment PK model with first-order absorption and a direct-response fractional Emax PD model. The initial run using this model failed to converge due to the small sample size, which did not support the covariate structure. It was decided to add the current data to the previous dataset to provide robust individual estimates.

### Classification of Histamine Phenotypes

Each response-time profile was assigned to one of three phenotype groups; hypo-, normo, or hyper-response based on pharmacodynamic parameters and visual inspection of individual predicted response vs. observed response plots as described previously (Jones et al., 2013). To objectively assign response phenotypes to these profiles, a method was developed using residuals (observed values – predicted values) derived from the pharmacometric analysis. For the profiles that could be assigned phenotype classification based visual inspection, the mean of the residuals for each profile was determined. The mean residuals for each profile were then plotted by phenotype group and examined. A tolerance level was then selected for the hypo- and hyper-responsive groups to reduce the amount of overlap with the normo-responsive group. The remaining response-time profiles were then assigned to a phenotype group based on the mean of residuals.

### Reliability of Histamine Phenotype Classification

The reliability of the histamine phenotype classification was determined for participants with two or more useable HILD profiles. The intraclass correlation coefficient (ICC) (Koo and Li, 2016) was calculated using the “ICC” package (v2.3.0) in R (v3.3.2). The ICC statistic can be used for test-retest purposes (Koo and Li, 2016).

## Results

### Demographics

Nineteen children with asthma and/or allergic rhinitis were enrolled for this study. Participants age ranged from 8 to 19 years; 64% were male. Most participants were Caucasian (36%) or African American (45%) (Table 1).

**TABLE 1:** Demographic characteristics of all participants with two or more HILD tests (*n* = 11).

Characteristic (*n* = 11)	Median (Range), % or N
Age (y)	11 (8–19)
BMI (kg/m^2^)	20.1 (15.8–26.4)
Sex (male)	64
Asthma diagnosis	27
**Race**	
Caucasian	4
African American	5
Asian	2

### Phenotype Response Classification

**FIGURE 2:**
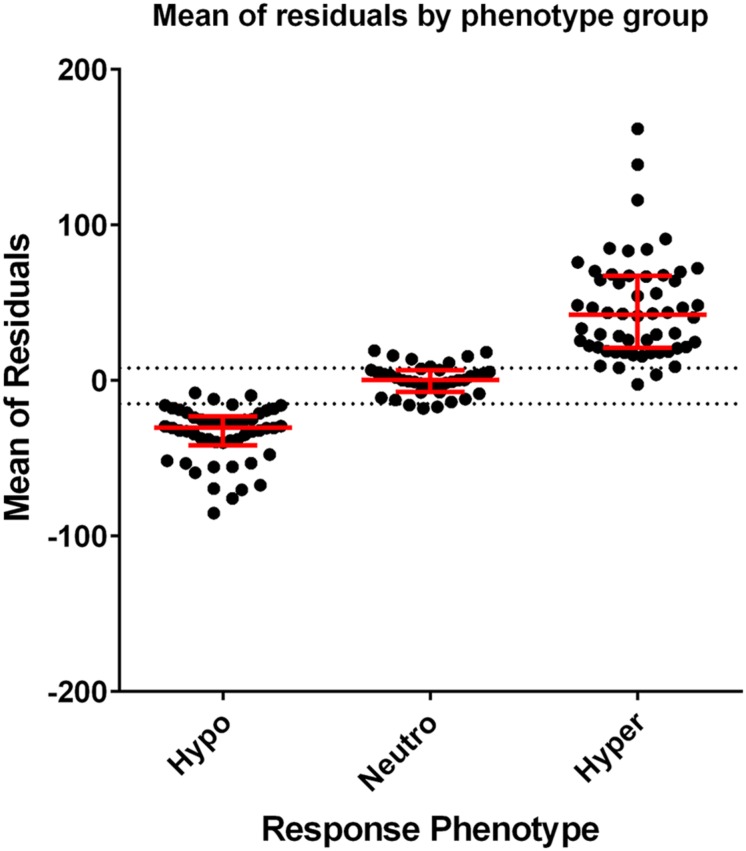
Scatterplots of the individual mean of residuals grouped by response phenotype. Dots represent the mean of residuals from each profile included in analysis. Red lines represent the median and interquartile range of the data. The dotted lines (–15 and +8) show the tolerance level of defining the response phenotype (mean of residuals > +8 is classified as hyper-responsive, mean of residuals < –15 is classified as hypo-responsive, and ≥ –15 or ≤ +8 is classified as normo-responsive).

Figure 2 demonstrates the distribution of mean residuals for the three phenotype groups. The median (range) for the mean of residuals were −30.5 (−85.3 to −7.98), 0.26 (−17.9 to 19.24), and 42.23 (−2.4 to 161.9) for the hypo-, normo-, and hyper-response phenotypes, respectively. A tolerance level of −15 was chosen for the hypo-responsive group, and a tolerance level of +8 was chosen for the hyper-responsive group. Values between these tolerance levels were considered as normo-responsive. Of the 41 profiles that were not originally assigned to a phenotype group, two were assigned to the hypo- phenotype, 24 to the normo- phenotype, and 15 to the hyper- phenotype using this method.

### Reliability of Phenotype Classification

**FIGURE 3:**
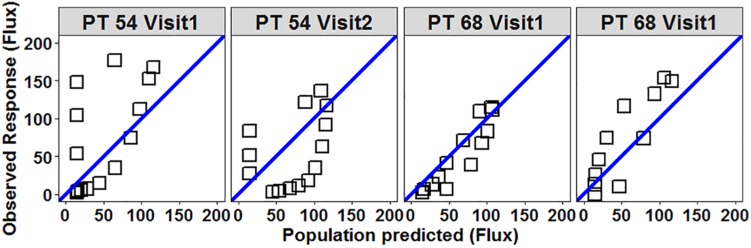
Scatterplot of observed vs. population predicted flux (response) for participants 54 and 68 demonstrate variability between measurements. Open squares represent individual flux values collected during each HILD measurement. Blue line is the line of unity. Participant 54 – on Visit 1 the mean of residuals was +26.5 (classified as hyper-responsive) and on Visit 2 the mean of residuals was –24.4 (classified as hypo-responsive). Participant 68 – on Visit 1 the mean of residuals was –7 (classified as normo-responsive) and on Visit 2 the mean of residuals was +20.8 (classified as hyper-responsive).

All four participants with four useable profiles had the same phenotype classification for each test. Five of the seven participants with two useable profiles had the same phenotype classification. One participant’s histamine response classification was initially hyper-response for the first test and subsequently was classified as hypo-response for the second test (Figure 3). The other participant had their histamine response classified as hypo-response on the first test and then hyper-response on the second test (Figure 3). The ICC of the HILD in this patient cohort was 0.77 (95% CI 0.44–0.93) suggesting a moderately reliable phenotype.

## Discussion

Histamine pharmacodynamic response phenotype as determined by HILD in children with allergic disease and/or asthma appears reliable based on our study findings. These data are essential as a next step in validating this potential biomarker in aiding therapeutic decision making in the use of anti-allergic/anti-histaminergic agents.

We have previously revealed that children with a hyper-responsive histamine response phenotype demonstrated higher AUEC, Emax, and Tmax values when compared to children classified as hypo-responsive phenotype based on observed vs. predicted plot visualizations (Jones et al., 2013). However, in this study, we also identified additional objective criteria to determine histamine response phenotype classification based on residual values, which is vital in the future evaluation of this biomarker to predict therapeutic response. Forty-one children were not able to be classified by observed vs. predicted plot visualization. As the majority of the observed vs. predicted plots were able to be clearly classified by visual inspection, there remains a segment of the profiles which are not easily discernable visually and therefore may lead to more subjective classification. A majority (58%) of these profiles were classified as normo-responsive by residual value classification. The residual value classification appears to provide an additional layer of objective classification, especially for non-readily apparent phenotypes and allow validation of visually observed plot phenotype classifications. This approach allows for improved phenotype classification reliability overall.

The moderate reliability of response phenotype classification in our study was likely partially due to our limited sample size. Although the majority of participants demonstrated reliable histamine response classification with repeat assessment, there were two participants whose classification changed from hyper-responsive to hypo-responsive and vice versa. Larger sample size would produce a more robust estimate of the confidence in the reliability of the phenotype classification via HILD. We did not identify the difference in phenotype reliability based on reported demographic characteristics. However, it is plausible that changes in underlying disease pathophysiology such as atopy and/or disease state (e.g., controlled vs. uncontrolled disease or allergic flare vs. stable allergic symptomatology) may be relevant to changing phenotype classification. These factors were not able to be considered in this study. Further investigation is required to determine the basis of response phenotype changes.

While HILD provides an objective, continuous, and dynamic measurement of histamine response compared to the epicutaneous histamine “skin prick” test, the method does have some limitations. Due to the sensitivity of microvascular measurements, the flowmetry is sensitive to movement. A majority of response-time profiles that were discarded were due artifact related to excessive movement during the procedure. Doppler flowmetry measurements were obtained post histamine administration in participants until flux values returned to pre-histamine baseline flux values or for a maximum duration of 2 h. During this time, the participant is made as comfortable as possible and is entertained with activities that require minimal movement (e.g., movie). However, the requirement for limited movement is a challenge, especially in the pediatric population. It may be possible to optimize further the method such that early data such as Emax and Tmax are sufficient to provide a phenotype classification which would be more ideal as a therapeutically useful biomarker.

## Conclusion

In conclusion, the data from the present study suggest that HILD has potential as a reliable and objective biomarker to describe histamine pharmacodynamic response in children. This is an essential next step in validating HILD in predicting clinical response to anti-allergic treatment. Future studies are also necessary in a larger sample size that considers the impact of changes in disease pathophysiology and/or disease state on pharmacodynamic response phenotype to further validate this technique and its utility in aiding therapeutic decision making for allergic/inflammatory diseases.

## Data Availability Statement

The datasets generated for this study will not be made publicly available. Consent was not obtained from the participates and their families for the data to be made available.

## Ethics Statement

The studies involving human participants were reviewed and approved by the Children’s Mercy Institutional Review Board (IRB # 11120477). Written permission assent to participate was provided by the participant’s legal guardian/next of kin.

## Author Contributions

BJ designed and managed the study, as the study PI. CS, SK, XL, and BJ cleaned and analyzed the data. All authors were involved in the drafting, editing and reviewing of the manuscript.

## Conflict of Interest

CS is Specialty Chief Editor in Frontiers Obstetric and Pediatric Pharmacology Journal. The remaining authors declare that the research was conducted in the absence of any commercial or financial relationships that could be construed as a potential conflict of interest.
